# *Cestrum nocturnum* Flower Extracts Attenuate Proliferation and Induce Apoptosis in Malignant Cells through Inducing DNA Damage and Inhibiting Topoisomerase II Activity

**DOI:** 10.1155/2017/1456786

**Published:** 2017-01-31

**Authors:** Deng-Pan Wu, Tian-Yu Lin, Jin-Yan Lv, Wen-Ya Chen, Li-Ru Bai, Yan Zhou, Jin-Lan Huang, Zhen-Guo Zhong

**Affiliations:** ^1^Jiangsu Key Laboratory of New Drug Research and Clinical Pharmacy, Pharmacy School of Xuzhou Medical University, Xuzhou, Jiangsu 221004, China; ^2^Department of Pharmacology, Pharmacy School of Xuzhou Medical University, Xuzhou, Jiangsu 221004, China; ^3^Faculty of Chinese Medicine Science, Guangxi University of Chinese Medicine, Nanning, Guangxi Zhuang Autonomous Region 530200, China; ^4^Department of Science and Technology, Guangxi University of Chinese Medicine, Nanning, Guangxi Zhuang Autonomous Region 530200, China

## Abstract

Most of the existing chemotherapeutic drugs have plenty of side effects. Chinese herbal medicine has been used for pharmaceutical and dietary therapy for thousands of years with more effective and fewer side effects.* Cestrum nocturnum* (CN) has long been used to treat digestive diseases for centuries in China. Our previous study first proved that the n-butanol part isolated from the flowers of CN produced an inhibitory effect on the proliferation of malignant cells. However, the fractions responsible for the antiproliferation effect of n-butanol part from CN flowers and related mechanisms remain unknown. Thus, in this study, we extracted fractions C4 and C5 from n-butanol part of CN flowers and investigated their immune toxicity and antitumor activities. It was found that fractions C4 and C5 exhibited great cytotoxicity to cancer cell lines but had low immune toxicity towards T and B lymphocytes in vitro. The tested fractions also attenuated proliferation and induced apoptosis at G_0_/G_1_ and G_2_/M phases in Bel-7404 cells through inducing DNA damage and inhibiting topoisomerase II relaxation activity. These results suggest that fractions C4 and C5 may represent important sources of potential antitumor agents due to their pronounced antitumor effects and low immune toxicity.

## 1. Introduction

Malignancy tumor has been ranked the fourth cause of death worldwide [1] and is responsible for about 1600 deaths everyday [2]. Although the methods of cancer diagnosis and therapy have made a rapid progress recently, the efficacy of cancer treatments has not been improved significantly yet. Currently, the prevailing approaches to treating malignant tumor include surgery and chemotherapy. However, most of chemotherapeutic drugs present plenty of side effects. Therefore, searching for safe and effective antitumor agents is the aim of current anticancer research.

*Cestrum nocturnum* (CN), a plant that belongs to the genus* Cestrum nocturnum* Linn., family Solanaceae, is widely distributed in Fujian, Guangdong, and Yunnan provinces as well as Guangxi Zhuang Autonomous Region, China. CN has long been used in traditional Chinese medicine (TCM) to treat digestive diseases for centuries [3]. Numerous studies have identified that CN has a great deal of pharmacological actions, including analgesic action, central inhibitory action, and antidiabetic activity [4–6]. Our previous study first proved that the n-butanol part isolated from the flowers of CN produced an inhibitory effect on the proliferation of human hepatocellular carcinoma Bel-7404, human gastric carcinoma SGC-7901, and cervical cancer HeLa cells in a dose-dependent manner [7]. However, the fractions responsible for the antiproliferation effect of n-butanol part from CN flowers and related mechanisms remain unknown.

Thus, this study was designed to evaluate the cytotoxicity of the fractions isolated from n-butanol part of CN flowers towards selected human malignant cell lines to determine the active fractions, elucidate the possible mechanisms of the cytotoxic effects of the tested fractions, and measure the immune toxicity of the fractions in vitro, so as to provide evidence for these fractions as potential antitumor agents.

## 2. Materials and Methods

### 2.1. Chemicals

The flowers of CN were collected in Guangxi Zhuang Autonomous Region, China, and verified by Professor Liu Shouyang from Guangxi University of Chinese Medicine. The voucher specimen (collection number: PE01621610) is deposited in Chinese Virtual Herbarium in Kunming, Yunnan province. Air-dried and powdered CN flowers (1.948 kg) were conducted with 95% and 50% ethanol (each 4000 mL) by percolating at room temperature overnight to give total ethanol extracts (424 g). The extracts were then mixed with silica gel thoroughly and sequentially partitioned with petroleum ether (2000 mL), acetic ether (2000 mL), water-saturated n-butanol (2000 mL), and 95% ethanol (2000 mL) to obtain four different parts. n-Butanol part (100 g) was subjected to macroporous resin column, eluting with water (1000 mL), 20% ethanol (1000 mL), 50% ethanol (1000 mL), 70% ethanol (1000 mL), and 95% ethanol (1000 mL) to give fraction B1 (20 g), B2 (14 g), B3 (29.5 g), B4 (6 g), and B5 (1 g), respectively. Using silica gel column chromatography, fraction B4 was partitioned with a gradient CHCl_3_ : CH_3_OH (30 : 1 to 1 : 1, each 1000 mL) and CHCl_3_ : CH_3_OH : water (65 : 35 : 10, 1000 mL) to give fraction C1~C10, respectively. The qualitative chemical profile of fractions C4 and C5 was analyzed by high-performance liquid chromatography (HPLC). The chemical formulas of compounds in fractions C4 and C5 were analyzed by single mass. In addition, the active ingredients in fractions C4 and C5 were analyzed by phytochemical analysis. HPLC, single mass, and phytochemical analysis were performed as described in the Chinese Pharmacopoeia (sixth edition).

### 2.2. Reagents

Wright's stain was obtained from SSS Reagent Co., Ltd., Shanghai, China. Giemsa's stain was from Chemical Reagent Factory, Shanghai, China. Agarose was from Bio-Science and Technology Limited Company, Shanghai, China. RNAse A was purchased from Shanghai Bio-Technology Limited, Shanghai, China. Low melting point agarose was from Dahui Bio-Technology Limited, Guangzhou, China. Propidium iodide (PI) and Hoechst 33342 were obtained from KeyGEN Bio-Technology Limited Company, Nanjing, China. pBR 322 DNA plasmid was from Takara Bio-Technology Limited Company, Dalian, China. Topoisomerase II*α* was purchased from KeyGEN Bio-Technology Limited Company, Nanjing, China. All other reagents were from Sigma-Aldrich unless stated otherwise.

### 2.3. Cell Culture and Treatment

Single T and B cell suspensions were isolated from the spleens of healthy Kunming mice and cultured in RPMI-1640 (Invitrogen, America) containing 10% fetal bovine serum (FBS, Hyclone, America). Human nasopharyngeal carcinoma CNE-2Z, human hepatoma Bel-7404, and human cervical cancer HeLa cell lines obtained from Shanghai Biological Institute were cultured in RPMI-1640 (Invitrogen, America) containing 10% FBS (Hyclone, America) in 5% CO_2_ at 37°C. The cell lines were divided into a control group and experimental groups treated with different concentrations of extracts from CN flowers.

### 2.4. Detection of Cytotoxicity by Methyl Thiazolyl Tetrazolium (MTT) Assay

CNE-2Z, Bel-7404, and HeLa cells were seeded to 96-well plates for 24 h. The cells of experimental groups were treated with fractions B1~B5, C1~C10, or 5-fluorouracil (5-Fu, positive control) at the concentrations of 5 *μ*g/mL, 10 *μ*g/mL, 20 *μ*g/mL, 40 *μ*g/mL, and 80 *μ*g/mL for 72 h, respectively. 0.2 mg/mL MTT solution was then added to incubate the cells for 4 h followed by removing the supernatant and adding 100 *μ*L of DMSO. Absorbance at 450 nm wavelength (*A*_450_) was detected using an enzyme-labeling instrument (Sunrise, Biocell). Inhibition rate (IR) = (1 − average *A*_450_ of the experimental group/the average *A*_450_ of the control group) × 100%.

### 2.5. Detection of Immune Toxicity by Cell Counting Kit-8 (CCK-8) Assay

2 × 10^6^ cells/mL single T and B cell suspensions were seeded into 96-well plates (100 *μ*L/well). After exposure to 5 *μ*g/mL Con A (Solarbio, China) and 10 *μ*g/mL LPS (Solarbio, China), T and B cells were treated with 5, 10, 20, 40, and 80 *μ*g/mL fraction C4 or C5 for 72 h, respectively. 10 *μ*L of CCK-8 (Dojindo, Japan) solution was added to incubate the cells for 1 h. Absorbance at 450 nm wavelength (*A*_450_) was detected by an enzyme-labeling instrument (Sunrise, Biocell). The inhibition rate (IR) = (1 − average *A*_450_ of the experimental group/the average *A*_450_ of the control group) × 100%.

### 2.6. Detection of Proliferation by Colony Forming Assay

200 cells/mL Bel-7404 cells were seed into 24-well plates overnight. Cells were treated with 10 *μ*g/mL fraction C4, C5, or 5-Fu (positive control). The cells were incubated for 7 days. During this period, each individual surviving cell would proliferate and form colonies. On Day 7, after the supernatant was removed, the colonies were stained with Wright's stain for 5 min followed by Giemsa's stain for 10 min. The colonies were then washed and dried. The colonies with >50 cells/colony were counted. The colony formation rate = the number of colony/the number of cells seeded into plate × 100%.

### 2.7. Detection of Cell Cycle by Flow Cytometry (FCM)

10^5^ cells/mL Bel-7404 cells were seeded into a 6-well plate for 24 h, and then the cells were treated with or without 10 *μ*g/mL fraction C4 or C5 for 72 h. The cells were collected and washed with PBS twice and fixed with cold 70% alcohol at 4°C for 24 h. After the alcohol was removed, the cells were then washed with cold PBS twice, exposed to 50 *μ*g/mL Rnase A at 37°C for 1 h, and stained with 10 *μ*g/mL PI and 2 mM Hoechst33342. Cell cycle and apoptosis were detected using FCM (BD, America).

### 2.8. Detection of DNA Damage by Alkaline Comet Assay

The alkaline comet assay was performed and modified following Singh protocol [8]. 10^6^ cells/mL Bel-7404 cells were seeded into a 6-well plate for 24 h followed by treatments with or without 40 *μ*g/mL fractions C4 and C5 for 72 h. The medium was removed and the cells were washed with ice cold PBS and trypsinized. 10^6^ cells/mL of cell suspension in PBS was mixed with 1% low melting point agarose (1 : 3, v/v) at 37°C, and then the suspension was pipetted onto slides precoated with normal melting point agarose. Slides with embedded cells in agarose were allowed to solidify at 4°C for 10 min. The slides were then placed for 40 min in a freshly prepared lysis buffer (2.5 M NaC1, 100 mM EDTA, 10 mM Tris, 1% Triton X-100, and 10% DMSO, pH 10). After lysis, the slides were placed in a horizontal electrophoresis chamber and incubated for 30 min at 4°C in an electrophoresis buffer (300 mM NaOH and 1 mM Na_2_EDTA, pH 13) for alkali DNA unwinding. Electrophoresis was performed in the same buffer for 40 min at 20 V and 240 mA at 4°C. After electrophoresis, the slides were rinsed three times with 0.4 M Tris-HC1 (pH 7.5) for 5 min and then stained with ethidium bromide (EB) (Beyotime, China). The comets were observed by fluorescent microscope (Nikon, Japan). Fifty cells were randomly selected per slide and the content of head and tail DNA in the comet was measured by comet assay software project (CASP). The percentages of DNA in the comet head (headDNA%) and tail (tailDNA%) were considered as parameters of the content of head and tail DNA, respectively.

### 2.9. Cleavage Assay of Topoisomerase II

This assay was performed according to Janočková et al. and Ketron et al. with modification [9, 10]. In the assay, plasmid PBR322 DNA containing supercoiled and linear DNA was used as the cleavage substrate of topoisomerase II. In brief, the reaction system was a 20 *μ*L reaction mixture containing 5 *μ*L reaction buffer (50 mM Tris-HCl, pH 7.5, 85 mM KCl, 10 mM MgCl_2_, 5 mM DTT, 30 g/mL BSA, 5 mM EDTA, and 1 mM ATP), 2 *μ*L plasmid PBR322DNA (final concentration: 10 *μ*g/mL), 10 *μ*L topoisomerase II*α* and 3 *μ*L fraction C4 (final concentration: 10, 20, or 40 *μ*g/mL), or C5 (final concentration: 10, 20 or 40 *μ*g/mL) or topoisomerase II inhibitor etoposide (Selleck Chemicals, America, final concentration: 100 *μ*M, positive control). The reactions were carried out for 30 min at 37°C and stopped by adding 10 *μ*L 10% SDS. Samples were digested by 10 mg/mL proteinase K (Beyotime, China) at 37°C for 30 min. Gel electrophoresis was performed at 50 V/cm for 1.5 h in a 0.5x TBE buffer containing 8.9 × 10^−2 ^M Tris, 8.9 × 10^−2 ^M H_3_BO_3_, and 2.0 × 10^−3 ^M EDTA on 0.8% agarose gel which was stained with 1 mg/mL EB. DNA bands were visualized using ultraviolet (UV) light (UVP, America). DNA cleavage was monitored by the conversion of supercoiled plasmid DNA to linear molecules. The intensity of linear DNA band in each lane was measured using imageJ software.

### 2.10. Statistical Analysis

Experimental data in each group were presented as mean ± SD. Analysis of variance was performed with SPSS software for windows 11.0. *P* < 0.05 was considered statistically significant.

## 3. Results

### 3.1. In Vitro Cytotoxic Activity

**Figure 1:**
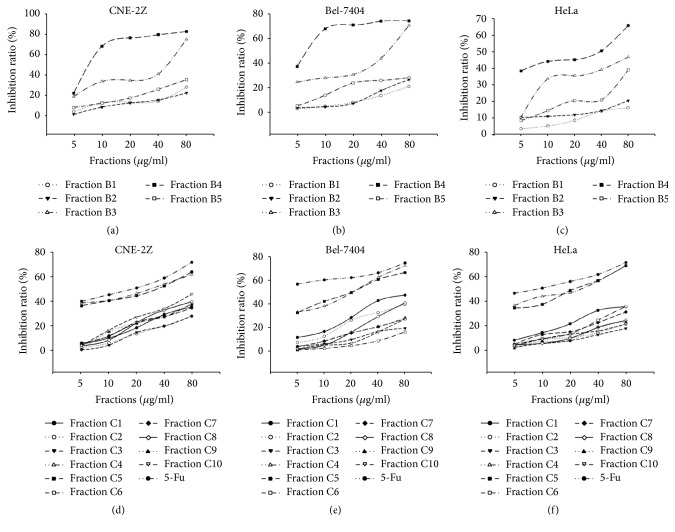
In vitro cytotoxicity of the extracts from CN flowers. MTT assay was used to estimate the cytotoxicity of the extracts to CNE-2Z, Bel-7404, and HeLa cells. Representative graphs are shown.

In order to measure the cytotoxicity of the fractions isolated from the n-butanol part of CN flowers, we first treated CNE-2Z, Bel-7404, and HeLa cells with different concentrations of fractions B1, B2, B3, B4, or B5 isolated from the n-butanol part of CN flowers for 72 h. MTT assay was used to measure the cytotoxicity of the fractions to these malignant cells. All fractions investigated exerted cytotoxic effects on the malignant cells in a dose-dependent manner. The increase in inhibitory efficacy induced by the five fractions of CN is shown in Figures 1(a), 1(b), and 1(c). It should be noted that fraction B4 exhibited the highest cytotoxic effects against the target malignant cells as shown by the corresponding inhibitory concentration 50% (IC_50_) in Table 1.

For further investigating effective antineoplastic fractions of CN flowers, fraction B4 was partitioned with a gradient CHCl_3_:CH_3_OH using silica gel column chromatography and ten fractions (from C1 to C10) were obtained. MTT assay showed that fractions C4 and C5 exited the greatest cytotoxicity to malignant cells as shown by the inhibition rate and the corresponding IC_50_ of target cells in Figures 1(d), 1(e), and 1(f) and Table 1, respectively.

### 3.2. Phytochemical Analysis, HPLC, and Single Mass Analysis of Fractions C4 and C5 of CN Flowers

**Figure 2:**
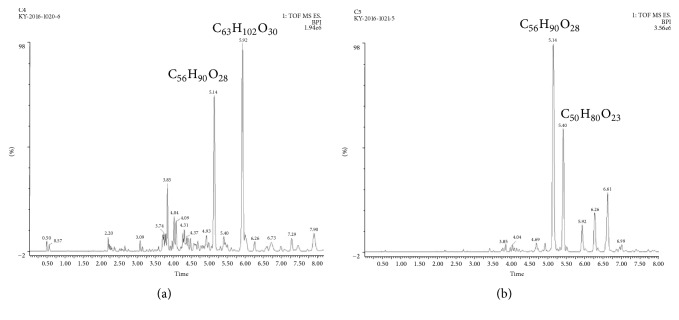
HPLC chromatograms of fractions C4 and C5. The chemical formulas of the main compounds in fractions C4 and C5 were analyzed using single mass analysis.

According to phytochemical analysis, steroidal saponins were found in fractions C4 and C5 since the appearance of green to pink color after the samples were treated with chloroform, acetic anhydride, and a series of concentrated HCl, and frothing bubbles were observed after vigorous shaking using diluted samples. HPLC and single mass analysis were adopted to identify the chemical profiles and formulas of compounds in fractions C4 and C5. Results showed that there were 2 main peaks in fractions C4 and C5 (Figure 2), and the chemical formulas of compounds were C_56_H_90_O_28_ (5.14 min) and C_63_H_102_O_30_ (5.92 min) in fraction C4 and C_56_H_90_O_28_ (5.14 min) and C_50_H_80_O_23_ (5.40 min) in fraction C5 (Figure 2).

### 3.3. Effects of Fractions C4 and C5 on Immune Toxicity

After treatment with different concentrations of fraction C4 or C5 for 72 h, CCK-8 assay was used to measure the proliferation of T and B cells to assess the immune toxicity of the fractions. The IC_50_ values of fraction C4 for T and B cells reached to 414.77 *μ*g/mL and 559.91 *μ*g/mL, respectively, and the IC_50_ values of fraction C5 for T and B cells were 543.31 *μ*g/mL and 244.41 *μ*g/mL, respectively, indicating that fractions C4 and C5 have low immune toxicity in vitro.

### 3.4. Effects of Fractions C4 and C5 on Colony Formation

**Figure 3:**
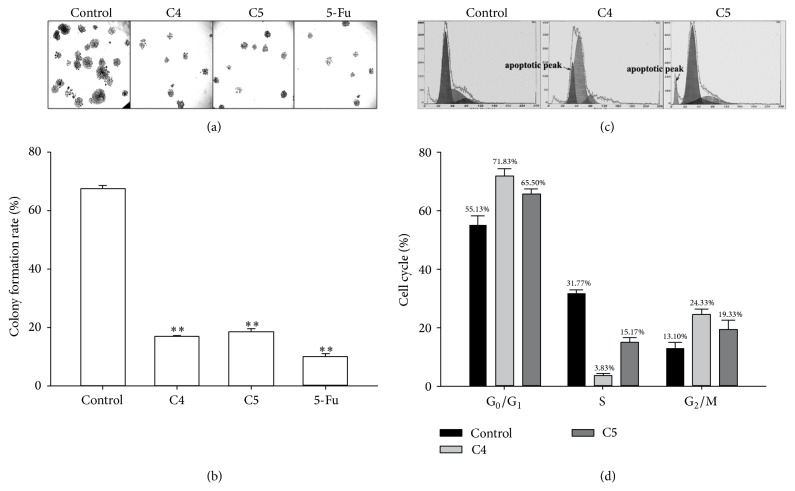
(a) and (b) Effects of fractions C4 and C5 on the colony formation of Bel-7404 cells. After the cells were treated with fraction C4, C5, or 5-Fu, the colonies (>50 cells/colony) were counted and the colony formation rate of each group was calculated. The data in different groups were expressed as the mean ± SD from 3 experiments. Statistical difference between groups was assessed by *t*-test using SPSS 20.0. ^*∗∗*^*P* < 0.01 versus the control group. (c) and (d) Flow cytometric analysis of the cell cycle distribution of Bel-7404 cells treated with fraction C4 or C5.

For further confirming the antineoplastic effects of fractions C4 and C5, colony forming assay was performed to determine the proliferation of Bel-7404 cells after exposure to 10 *μ*g/mL of fraction C4, C5, or 5-fluorouracil (5-Fu) for 7 days. Results revealed that the colony formation rate of the cells treated with fraction C4 or C5 was remarkably decreased compared with the control cells (Figures 3(a) and 3(b)), suggesting that the proliferation of Bel-7404 cells is attenuated by fractions C4 and C5.

### 3.5. Analysis of Changes in Cell Cycle Phase Distribution

The effects of fractions C4 and C5 on Bel-7404 apoptosis and cell cycle distribution were measured by flow cytometry. As shown in Figures 3(c) and 3(d), treatment with 10 *μ*g/mL fraction C4 or C5 for 72 h resulted in an increase in the number of the cells at the G_0_/G_1_ and G_2_/M phases whereas a decreased number of the cells at the S phases compared with the control cells and an apoptotic peak were observed after treatment with fraction C4 or C5. These results indicate that fractions C4 and C5 arrest the cells at the G_0_/G_1_ and G_2_/M phases and cause apoptosis.

### 3.6. Detection of DNA Damage Using Alkaline Comet Assay

**Figure 4:**
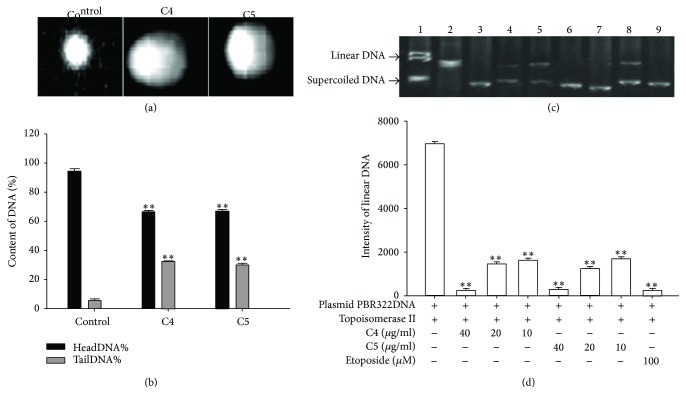
(a) and (b) DNA damage induced by fractions C4 and C5 in Bel-7404 cells measured by comet assay. Fifty cells were randomly selected per slide and the contents of head and tail DNA in the comet were measured by comet assay software project (CASP). The percentages of DNA in the comet head (headDNA%) and tail (tailDNA%) were considered as parameters of the content of head and tail DNA, respectively. The data in different groups were expressed as the mean ± SD from 3 experiments. Statistical difference between groups was assessed by *t*-test using SPSS 20.0. ^*∗∗*^*P* < 0.01 versus the control group. (c) and (d) Effects of fractions C4 and C5 on topoisomerase II activity. Topoisomerase II activity was measured by plasmid DNA cleavage assay. DNA bands were visualized using UV light and the intensity of linear DNA band in each lane was measured using imageJ software. Lane 1: plasmid PBR322DNA. Lane 2: control, topoisomerase II + plasmid PBR322DNA. Lanes 3, 4, and 5: 40, 20, and 10 *μ*g/mL fraction C4 + plasmid PBR322DNA, respectively. Lanes 6, 7, and 8: 40, 20, and 10 *μ*g/mL fraction C5 + plasmid PBR322DNA, respectively. Lane 9: 100 *μ*M etoposide + plasmid PBR322DNA. The data in different groups were expressed as the mean ± SD from 3 experiments. Statistical difference between groups was assessed by *t*-test using SPSS 20.0. ^*∗∗*^*P* < 0.01 versus the control group.

Comet assay is generally used to measure single and double-strand breaks, DNA repair, alkali labile sites, and DNA-protein/DNA-DNA/DNA-agent cross linkage [11]. The principle of the assay depends on strand breakage of the supercoiled double helical DNA, resulting in the reduction in the size of large molecules. Comets form when these broken ends of the negatively charged DNA molecule can migrate freely towards the anode in the electric field [12]. If DNA breakage were induced, the percentage of DNA in the comet head would decline; in contrast, the percentage of DNA in the comet tail would increase. In the present study, DNA damage of Bel-7404 cells was assessed using comet assay after these cells were treated with 40 *μ*g/mL fraction C4 or C5 for 72 h. As shown in Figures 4(a) and 4(b), the comets of fraction C4 or C5 treated cells were clearly visible under the microscope. However, no comet formation was observed within untreated cells. In addition, the percentage of head DNA in fraction C4 or C5 treated cells was remarkably lower than the control group. In contrast, the percentage of tail DNA that revealed the actual DNA damage, in the cells which are exposure to the studied fractions, was significantly increased compared to the control group. Specifically, the percentages of tail DNA in fraction C4 and C5 treated cells were approximately 33% and 30%, respectively, whereas the percentage of tail DNA in control group was 6%, so fractions C4 and C5 increased the contents of tail DNA by a factor of approximately 5. These results indicate that fractions C4 and C5 have abilities to induce DNA damage.

### 3.7. Effects of Fractions C4 and C5 on DNA Relaxation Activity of Topoisomerase II

It has been established that topoisomerase II relaxes the helical supercoiling of DNA that is generated during replication, chromatin remodeling, and transcription, leading to the conversion of supercoiled DNA to linear molecules [13]. In order to investigate the effects of fractions C4 and C5 on topoisomerase II relaxation activity, plasmid DNA cleavage assay was performed in the study. This assay is a specific reaction of topoisomerase II involving the simultaneous cleavage of supercoiled DNA. If the relaxation activity of topoisomerase II were allowed to continue unimpeded, supercoiled DNA would be cleaved by topoisomerase II and replaced by linear forms of DNA. On the contrary, if the enzymatic activity of topoisomerases II were suppressed, these linear DNA bands would not be detected. As illustrated in Figures 4(c) and 4(d), fractions C4 and C5 could reduce the intensity of linear DNA band in a dose-dependent manner, suggesting that fractions C4 and C5 have abilities to inhibit the DNA relaxation activity of topoisomerases II. Fractions C4 and C5 at 40 *μ*g/mL could completely inhibit the relaxation activity of topoisomerases II in a way similar to that of a known topoisomerase II inhibitor, etoposide (100 *μ*M) since linear DNA bands in these groups were rarely detected (Figures 4(c) and 4(d)).

## 4. Discussion

Chemotherapy, radiation, and surgery are the most paramount strategies used for the treatment of malignant tumors. However, these treatments exert various side effects. Chinese herbal medicine has been used for pharmaceutical and dietary therapy for thousands of years with more effective and fewer side effects. Therefore, developing safe and highly effective anticancer herbal agents have gradually become a hot area. In this study, we prepared different fractions from the n-butanol extract of CN flowers and measured their cytotoxicity to malignant cells using MTT assay. It was found that fractions C4 and C5 isolated from the n-butanol fraction of CN flowers had pronounced cytotoxic activities against CNE-2Z, Bel-7404, and HeLa cells (Figures 1(d), 1(e), and 1(f)). The results of phytochemical analysis showed that the tested fractions were mainly composed of steroidal saponins. The findings of HPLC and single mass analysis revealed that the compounds, with the chemical formulas of C_56_H_90_O_28_ (5.14 min), C_63_H_102_O_30_ (5.92 min), and C_50_H_80_O_23_ (5.40 min), accounted for a large proportion of fractions C4 and C5 (Figure 2), suggesting that these compounds may belong to steroidal saponins. Since most steroidal saponins were reported to produce great cytotoxicity towards cancer cells [14], the indicated compounds may exert antineoplastic activity and make contribution to the cytotoxicity of the tested fractions to malignant cells. It would be interesting to identify the chemical structures of these compounds and investigate their antitumor activities in the future study.

Increasing the antitumor activity of existing anticancer drugs, rather than enhancement of its toxicity, is the aim of current anticancer research. In our study, CCK-8 assay was used to investigate the immune toxicity of fractions C4 and C5 towards T and B lymphocytes. The results showed that the IC50 values of fractions C4 and C5 for T cells reached to 414.77 *μ*g/mL and 543.31 *μ*g/mL, respectively, while the IC50 values of fractions C4 and C5 for B cells were 559.91 *μ*g/mL and 244.41 *μ*g/mL, respectively, indicating that fractions C4 and C5 have mild immune toxicity in vitro.

Evaluation of the proliferation of cells is a noteworthy step since most of anticancer drugs are less cytocidal to quiescent cells than to actively proliferating cells [15]. In order to further assess the antitumor activities of fractions C4 and C5, colony forming assay was performed to determine the role of the tested fractions in the proliferation of Bel-7404 cells. The results revealed that treatment with fractions C4 and C5 exhibited an inhibitory effect of colony formation (Figures 3(a) and 3(b)), indicating that fractions C4 and C5 possess the abilities to attenuate the proliferation of Bel-7404 cells.

Apoptosis is the physiological process that contributes to elimination of unnecessary and unwanted cells to maintain the healthy balance between cell survival and cell death [16]. Abnormal apoptosis plays important roles in tumorigenesis and tumor progression. Inducing tumor cell apoptosis to annihilate tumor cells is one of the mechanisms of antineoplastics. In the present study, an apoptotic peak was observed after treatment with 10 *μ*g/mL fraction C4 or C5 for 72 h (Figures 3(c) and 3(d)), indicating that fractions C4 and C5 have abilities to induce apoptosis of Bel-7404 cells. Since abnormal cell cycle is known to be responsible for initiating apoptosis under cell damage conditions, cell cycle was detected with a flow cytometer 72 h after treatment with 10 *μ*g/mL fraction C4 or C5 in our study. The results demonstrated that treatment with fraction C4 or C5 resulted in the cells arrested at the G_0_/G_1_ and G_2_/M phases, suggesting that apoptosis induced by fractions C4 and C5 mainly occurs at the G_0_/G_1_ and G_2_/M phases (Figures 3(c) and 3(d)).

Apoptotic cells exhibit several biochemical modifications such as protein cross-linking, protein cleavage and DNA denaturation that ultimately lead to remarkably structural and pathological changes [17]. The alkaline comet assay is widely used for assessment of the genotoxicity of DNA damaging agents in vitro or in vivo [18]. In our study, DNA damage was detected using the alkaline comet assay after Bel-7404 cells were exposed to 40 *μ*g/mL fraction C4 or C5 for 72 h. The results showed that the comets of cells treated with fraction C4 or C5 were clearly visible under the microscope. However, no comet formation was observed in untreated cells (Figure 4(a)). The percentage of tail DNA in the fractions C4 and C5 treated cells was significantly increased compared to the controls, indicating the genotoxic property of fractions C4 and C5 (Figure 4(b)).

Topoisomerases are essential nuclear enzymes that are involved in DNA supercoiling regulation and play key roles in chromosome condensation, RNA transcription, DNA replication and segregation during mitosis, and attachment of DNA loops to the chromosomal scaffold and nuclear matrix [19]. Topoisomerases has two major classes, types I and II. They are distinguished by the number of DNA strands which they cleave and the mechanism by which they alter the DNA topological properties [20]. Topoisomerase II catalyzes an ATP-dependent reaction in which one DNA double helix is supercoiled through another [21]. Disruption of the enzymatic activity of topoisomerases II induces double-strand breaks (DSBs), triggering the DNA damage response (DDR) and subsequently causing apoptosis [22]. In our present study, it was found that fractions C4 and C5 could strongly inhibit the relaxation activity of topoisomerase II in a dose-dependent manner (Figures 4(c) and 4(d)), suggesting that inhibition of topoisomerases II relaxation activity may be one of the mechanisms of fraction C4- or C5-induced apoptosis. It is essential to elucidate further mechanisms of the inhibiting effect of the tested fractions on topoisomerase II activity and identify the role of the studied fractions in topoisomerase I activity in the future, since topoisomerase I is also considered as a DNA manipulating enzymes responsible for the breakage of one strand DNA [20].

## 5. Conclusion

Our results indicate that fractions C4 and C5 extracted from the n-butanol part of CN flowers showed significant cytotoxic potential towards a wide range of human malignant cell lines with low cytotoxicity to immunocytes and exhibited strong antitumor activities against Bel-7404 cells. These antitumor activities include attenuation of cancer cell proliferation as well as induction of apoptosis at the G_0_/G_1_ and G_2_/M phases through enhancement of DNA damage and inhibition of topoisomerase II relaxation activity. The pronounced antitumor actions and low immune toxicity of fractions C4 and C5 suggest that further studies are strongly needed to identify the chemical structures of the active compounds in these fractions and elucidate the antitumor activities of these compounds for their possible use as antitumor agents.

## Figures and Tables

**Table 1:** The concentrations of the CN flower fractions, which induced 50% decrease in malignant cells survival, according to MTT assay.

Fractions	IC_50_ (***μ***g/mL)		
	CNE-2Z	Bel-7404	HeLa
B1	411.71	440.11	845.9
B2	213.51	281.01	933.58
B3	34.42	37.23	73.87
B4	17.50	11.08	23.59
B5	202.1	201.13	181.70
C1	130.33	75.9	150.78
C2	162.53	109.49	909.41
C3	403.13	205.30	550.77
C4	19.78	18.71	19.21
C5	22.98	19.62	19.70
C6	211.10	360.32	144.68
C7	588.06	222.92	211.01
C8	387.63	113.80	335.91
C9	245.55	109.38	199.93
C10	272.11	201.4	452.3
5-Fu	16.43	2.32	8.96

The cells were exposed to the agents for 72 h.
